# Experimental investigation on solidification cracking & intergranular corrosion of AISI 321 & AISI 316 L dissimilar weld on pulsed current gas tungsten arc welding (PCGTAW)

**DOI:** 10.1016/j.heliyon.2024.e34648

**Published:** 2024-07-22

**Authors:** Tejas Patil, Ajit Bhosale, S.G.K. Manikandan, Bibin Jose, Mithul Naidu, Sachin Salunkhe, Robert Cep, Emad Abouel Nasr

**Affiliations:** a Department of Mechanical Engineering, Zeal College of Engineering and Research, Pune, India; b Department of Mechanical Engineering, Cummins College of Engineering for Women, Pune, India; c Welding and Coating Facilities, Indian Space Research Organization, Mahendragiri, India; d Department of Mechanical Engineering, Muthoot Institute of Technology & Science, Kochi, India; e Department of Mechanical Engineering, Deogiri Institute of Engineering & Management Studies, Chh. Sambhajinagar, India; f Faculty of Mechanical Engineering, Tolani Maritime Institute, Pune, India; g Department of Biosciences, Saveetha School of Engineering, Saveetha Institute of Medical and Technical Sciences, Chennai, India; h Gazi University Faculty of Engineering, Department of Mechanical Engineering, Maltepe, ANKARA, Turkey; i Department of Machining, Assembly and Engineering Metrology, Faculty of Mechanical Engineering, VSB-Technical University of Ostrava, 17. Listopadu 2172/15, 708 00, Ostrava, Czech Republic; j Department of Industrial Engineering, College of Engineering, King Saud University, P.O. Box 800, Riyadh, 11421, Saudi Arabia

**Keywords:** Dissimilar metal, ASS 316L, ASS 321, Inter-granular corrosion, Suutula diagram

## Abstract

Dissimilar metal combinations are frequently employed in the power generation and nuclear industries. Where stainless steel piping systems are connected to pressure vessels made of low-alloy steel, the subsystems of liquid rocket engines also have different, dissimilar material combinations. Dissimilar welding plays a vital role in ensuring the integrity, performance, and reliability of components and structures operating in cryogenic environments, in this study, plates of AISI 316L and AISI 321, each 5 mm thick, were successfully joined using the pulsed current gas tungsten arc welding (PCGTAW) technique with optimized process parameters. These weld joints are mostly present in rocket engines subjected to a cryogenic environment. Due to the low temperature environment, the metallurgical properties of these joints change, which affects their mechanical properties. As it is a structural part, PCGTAW welding is most common method for joining this kind of material.

In this work, Microstructural analysis of the weldment revealed a combination of vermicular, lacy, and acicular ferrite morphologies in the fusion zone at the root, mid, and crown locations.

Furthermore, no solidification cracking was detected in the weldments based on the optical micrograph and SEM results. Intergranular corrosion (IGC) testing indicated the absence of a ditch structure, suggesting that the heat-affected zone (HAZ) on both sides of the weld joint was not being susceptible to sensitization. However, the HAZ of the AISI 316L side exhibited coarser grains compared to AISI 321. Analysis of tensile properties revealed a significant influence of the testing environment on the tensile strength of the dissimilar welded joints. At room temperature, the average ultimate tensile strength (UTS) was measured as 621 MPa. Remarkably, at cryogenic conditions, the average tensile properties significantly increased to 1319 MPa.

Microhardness analysis showed the highest hardness associated with the AISI 321 side. The fusion zone exhibited a large deviation in the hardness profile (205 ± 10 HV), with the highest average hardness observed in the middle part of the weld. However, the hot cracking behavior of the weld was investigated by using a suutula diagram at various locations of the weld. The investigation revealed that the Cr_eq_/Ni_eq_ ratio exceeded the critical threshold value, effectively diminishing the propensity for hot cracking in the fusion zone. Overall, these findings underscore the effectiveness of the PCGTAW technique in joining dissimilar materials, as well as the importance of microstructural and mechanical property evaluations, especially under extreme operating conditions such as cryogenic temperatures.

Paraphrase.

## 1 Introduction

**Fig. 1:**
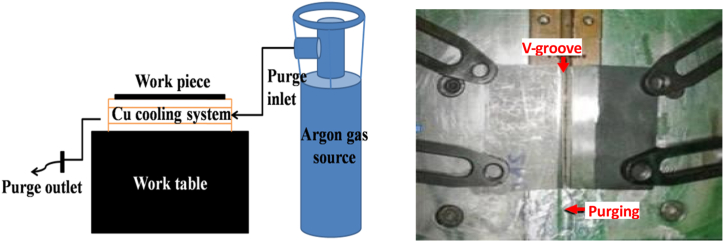
Welding setup.

Welding has been the foundation of modern industrial processes, playing a vital role in the fabrication of various components across diverse applications [[1], [2], [3], [4]]. In numerous engineering fields, dissimilar welding, a process where two distinct metals or alloys are joined together, offers unprecedented advantages in terms of optimizing material properties and meeting specific performance requirements [[5], [6], [7]]. Dissimilar metal welding not only meets the requirements of the service but also generates significant savings by reducing the amount of expensive material required in these joints [8]. Due to the different chemical compositions and coefficients of thermal expansion of the base metals used, welding dissimilar metals is a more cumbersome and challenging task than welding similar metals [9,10] (see Fig. 1).

**Fig. 2:**
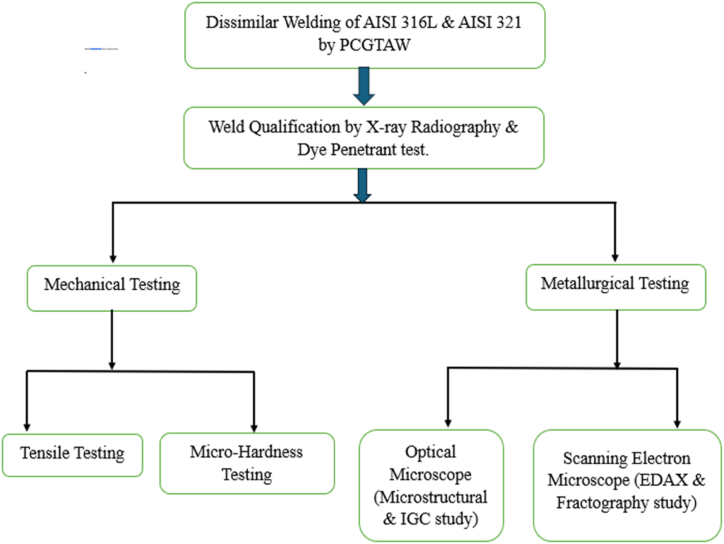
Methodology.

Austenitic stainless steels are easily weldable if appropriate precautionary measures are followed. The elements that stabilise the phase of austenite in the matrix are nickel, carbon, and nitrogen [[11], [12], [13]]. Mostly nickel is added to these steels in large amounts, whereas nitrogen and carbon are strong austenite promoters [14]. Dissimilar welding of different grades of austenitic stainless steel finds applications in various industries due to its unique advantages. This process involves joining distinct stainless-steel grades, offering flexibility in material selection for different components. Applications span from chemical processing to aerospace, where corrosion resistance and mechanical properties are critical [9,10]. The advantages include tailored material choices for specific environments, cost-effectiveness through reduced material usage, and the ability to create complex structures with desired characteristics. Arivarasu et al. [15] performed dissimilar welding of AISI 4340 and AISI 304L using autogenous PCGTAW. The study found that martensite microstructure was formed in the weld fusion zone. The study also revealed that AISI 4340 HAZ contains dispersed carbides, resulting in higher hardness. The dissimilar weldment showed elevated hardness and reduced impact toughness, with fractures consistently observed in the AISI 304L parent metal in all room-temperature trials. The average tensile strength was 708 MPa (see Table 4). However, The detailed methodology for the current study given below (see Fig. 2)

**Table 1:** Chemical Composition of Base and filler metal.

Elements	AISI316L	AISI321	Filler Metal
**C**	0.03	0.08	0.04
**Ti**	–	0.36	–
**V**	–	–	–
**P**	0.045	0.045	–
**S**	0.03	0.003	–
**Ni**	11.19	10.2	11
**Mo**	2.28	–	3
**Cr**	17.15	17.4	19
**Mn**	2	2	–
**Ti**	–	0.36	–
**Fe**	Bal	Bal	Bal

**Table 2:** Optimized Process parameters.

No of passes	Peak Current (A)	Average Current (A)	Base Current (A)
1	160	120	80
2	120	90	60
3	120	90	60
4	120	90	60

**Table 3:** Cr_eq_ to Ni_eq_ levels in the different zones of the fusion zone.

Sr.No	Location	Cr_eq_	Ni_eq_	Cr_eq_/Ni_eq_
1	Root	22.7153	11.4981	1.975
2	Middle	22.773	11.3477	2.006
3	Crown	22.7984	11.5474	1.974

**Table 4:** Tensile properties of the weldments in ambient and Cryogenic conditions.

Sno.	Condition	UTS (MPa)	0.2 % YS (MPa)	%Elongation
1	Room Temperature	621	326	45
2	Room Temperature	615	340	54
3	Room Temperature	626	345	42
Average		**621**	**337**	**47**
1	Cryogenic Temperature	1406	429	36
2	Cryogenic Temperature	1284	421	40
3	Cryogenic Temperature	1268	459	42
Average		**1319**	**436**	**39**

Acar et al. [16] investigated the weldability of dissimilar stainless steels (AISI 316 and AISI 430) using GMAW with varying gas combinations. It explores the microstructural evolution and mechanical properties of the welded joints. The study highlights the influence of welding parameters and shielding gas on the quality of the joints. Insights from the research contribute to enhancing the understanding of welding dissimilar stainless steels for industrial applications. Abhishek et al. [17] discuss the optimization of welding parameters for rail car bracket assembly using the Taguchi approach and RSM. It evaluates the effectiveness of the process in terms of hardness and distortion, highlighting the interaction of input components. The study also includes an ANOVA and regression analysis to validate the proposed model. Additionally, the research emphasizes the importance of optimizing welding factors such as voltage, current, welding speed, and arc length for efficient welding processes.

When compared to other stainless-steel grades, AISI 316L has the typical advantage of not being susceptible to sensitization at high temperatures [18]. AISI 321 contains titanium, which stabilizes the alloy against the development of chromium-rich carbides; it combines with carbon and reduces the tendency for intergranular corrosion caused by chromium carbide precipitation [19]. By combining these two distinct stainless-steel grades through welding, engineers can achieve a synergistic effect, potentially enhancing overall mechanical properties and structural integrity for critical components. Furthermore, AISI 316L and AISI 321 weldments are valuable in cryogenic engines [20]. Their weld properties ensure structural integrity and corrosion resistance at extremely low temperatures, enhancing performance and reliability in cryogenic applications [21].

However, welding AISI 316L and ASS 321 together presents its own set of challenges, such as the formation of intermetallic phases, thermal stresses, and possible reductions in corrosion resistance at the weld interface [22]. Additionally, choosing a suitable filler wire is the next challenge during dissimilar metal welding. Instances like weld solidification cracking, liquidation cracking at the HAZ, and many more metallurgical issues can result from incorrect filler wire selection [23]. According to reports, several welding defects, such as segregation, secondary phase formation, dilutions, and cracks, were developed in the weldments if the filler wire choice and welding procedure were not appropriate. Shojaati et al. [24] performed dissimilar welds of AISI 304 and AISI 409 using GTAW with four different filler wires (ASS 310, ASS 316L, duplex steel, and a nickel-based alloy).

The austenitic filler wire resulted in various ferrite morphologies, including vernicular, lathy, and acicular structures. The duplex steel filler wire exhibited a combination of austenite and ferritic structure, with widmanstatten morphology of austenite observed. The nickel-based filler wire contained a Ni, Cr, and Fe matrix with iron precipitates. The use of the duplex filler wire produced a high-strength weld with a substantial amount of delta ferrite in the microstructure, contributing to the increased hardness and tensile strength of the weld metal. During the welding of dissimilar metals, elemental migration is another major concern, which often affects the mechanical, metallurgical, and corrosion properties of the dissimilar weldments [25]. Therefore, it is crucial to exercise caution when choosing filler materials for dissimilar metal joining, focusing on mechanical properties like thermal, fatigue resistance, toughness, and hot cracking tendency.

A study conducted by Unnikrishnan et al. [26] investigates the impact of heat input on the microstructure, residual stresses, and corrosion resistance of 304L austenitic stainless-steel weldments using SMAW. The study addresses the sensitization phenomenon in stainless steels, highlighting the importance of maintaining corrosion resistance and mechanical properties in high-temperature applications. It was found that even at high heat inputs, SMAW of 304L stainless steel did not result in significant precipitation of carbides or intermetallic phases. The ferrite content and grain size increased with the rise in heat input. Additionally, the ferrite number (FN) and hardness of the welded region were measured, showing an increase in FN with heat input while hardness decreased due to the higher ferrite content in the weld metal.

This research paper aims to address the critical knowledge gap concerning the dissimilar welding of AISI 316L and AISI 321 stainless steels. By conducting a systematic study, we aim to shed light on the microstructural characteristics, mechanical properties, and corrosion behaviour of the welded joint, particularly for cryogenic applications. The outcomes of this research are expected to not only contribute to the understanding of dissimilar welding of stainless steels but also to offer valuable insights for engineering practitioners seeking reliable and efficient methods to weld AISI 316L and AISI 321 for demanding applications. The study also investigated the weld's hot cracking behaviour using the suutula diagram at different locations, revealing Cr_eq_/Ni_eq_. Ultimately, a comprehensive investigation of this dissimilar welding combination will pave the way for further advancements in material joining techniques, leading to safer and more robust engineering solutions across multiple industries.

## 2 Experimental setup

The 1.6 mm ER 316L filler metal was used to join the annealed 5 mm AISI 316L and AISI 321 plates together using PCGTAW with the optimized process parameters, and the welding was carried out in the presence of argon gas [10]. The chemical compositions of the filler metal and base metal are listed in Table 1. However, Table 2 shows the optimized welding parameters used for the present study. Furthermore, X-ray radiography and dye penetrant testing were used for the qualification of the weld, and they were done on welded samples [19]. Microstructural, EDAX and Intergranular corrosion (IGC) studies have been carried out on welded samples after specimen preparation to understand fusion zone metallurgical characteristics after electrolytically etching with 10 % oxalic acid.

For an intergranular corrosion study for the detection of susceptibility to intergranular attack in austenitic stainless steels on both sides of HAZ, the standard ASTM A262 Practice A was utilized. The weld specimen is immersed in a solution of 10 % oxalic acid at 675 °C for 1 h. After testing, the specimen is examined for signs of intergranular attack (IGA) or corrosion at the grain boundaries with microscopic examination at 250×. The results obtained were compared with the standard microstructures as given in the standard to assess the sensitization of the grain boundaries on both sides of the HAZ.

Furthermore, the samples were taken out of the weld pad for tensile tests at room and cryogenic temperatures. Since dissimilar combinations were involved at cryogenic temperatures, the tensile samples were made according to ASTM E1450. However, the ASTM E8 standard were followed for ambient condition [15]. To study the strength variation Vickers microhardness analyses were performed to assess the hardness profile across the weldments [10].

## 3 Result & discussion

### 3.1 Metallographic & EDAX analysis

To study the metallurgical behavior of the fusion zone and HAZ, optical and scanning electron microscopic methods were utilized. Microstructural studies were conducted in the fusion zone and heat-affected zone (HAZ) to investigate ferrite morphology. Efforts were also made to determine the critical Cr_eq_ and Ni_eq_ values within the fusion zone, with the objective of assessing the potential susceptibility of the joint to solidification cracking.

**Fig. 3:**
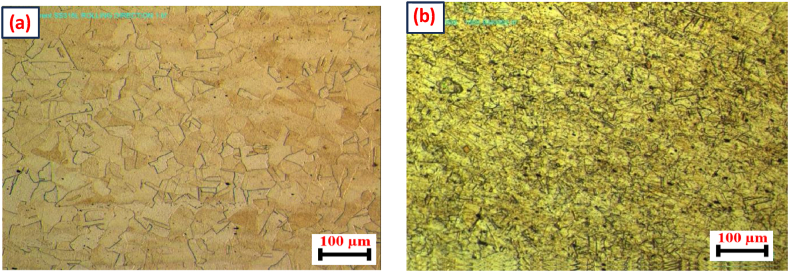
Optical micrograph of the base materials (a) AISI 316L (b) AISI 321.

Fig. 3 (a, b) depicts an optical micrograph of the base materials. The AISI 321 microstructure shows a fine grain-twinned austenitic structure with few carbides; it confines germanium or titanium carbide. Previous studies have noted similar findings, identifying cubic-shaped precipitates rich in titanium within the austenitic matrix [27]. Conversely, AISI 316L revealed a completely austenitic structure consisting of fine grain twins with properly defined grain boundaries. Generally, cold working is one of the strengthening mechanisms used to strengthen the matrix for AISI 321 and AISI 316L [27]. The addition of titanium minimizes the precipitation of chromium carbides along grain boundaries and reduces the effect of intergranular corrosion through very stable titanium carbide.

**Fig. 4:**
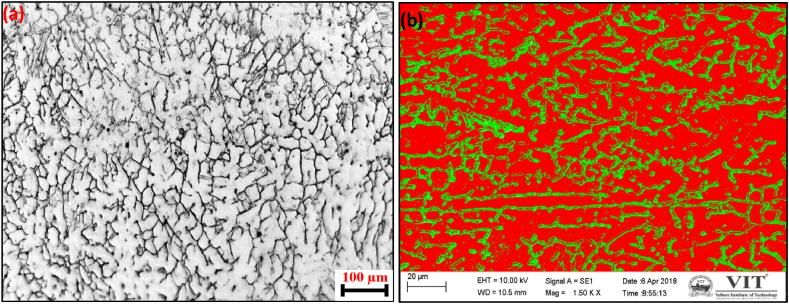
Optical (a) and SEM (b) images of root region.

**Fig. 5:**
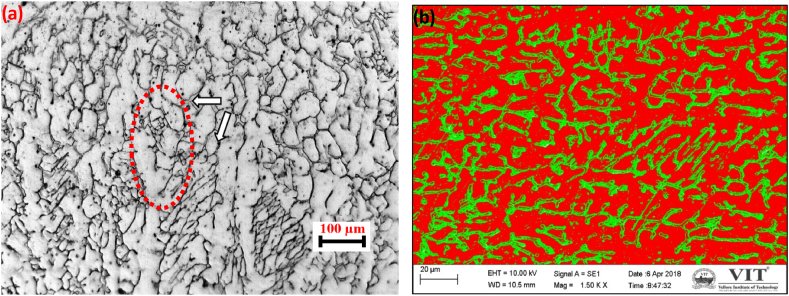
Optical (a) and SEM (b) images of mid region.

**Fig. 6:**
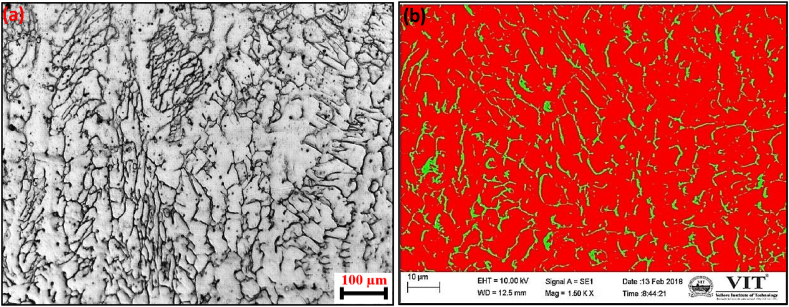
Optical (a) and SEM (b) images of crown region.

The room-temperature structure of the fusion zone of austenitic stainless steel is determined by both the solidification modifications and subsequent solid-state transformation. Depending on its unique composition, namely the Cr_eq_/Ni_eq_ ratio, all types of stainless steel solidify as either primary ferrite or primary austenite. However, small modifications in the alloy system composition may promote a transition from primary ferrite to primary austenite. It was found in the current work that primary ferrite had formed in the fusion zone, and whatever austenite was present in the inter-dendritic region of ferrite that could have formed at the end of solidification typically forms via a peritectic-eutectic reaction [7]. However, the ferrite morphology in the fusion zone depends on solidification, transformation sequence, and weld cooling rate. The microstructure of the fusion zone is divided into three parts, namely the root, middle, and crown parts, to understand the ferrite morphology of the weld (see Figs. 4–6).

Ferrite morphology investigations were conducted in the weld's root region at different locations. Fig. 4 shows the optical and SEM images of root regions. In the SEM images, the red color represents the austenitic matrix, and the green color depicts the ferritic. The results consistently revealed a vermicular ferrite morphology across all weld locations in the root region, as depicted in Fig. 4. This specific vermicular pattern typically arises when there is a low Cr_eq_/Ni_eq_ ratio and moderate rates of weld cooling. Fig. 5 shows the optical and SEM images of the mid-region.

The microstructure of the mid-region of the weld shows lacy ferrite morphology. This kind of morphology is found when the weld cooling rate is high and when the Cr_eq_/Ni_eq_ ratio increases in a particular location. Fig. 6 shows the optical and SEM images of crown regions. A micrograph of the crown part of the weld shows non-uniformly distributed ferrite stringers or needles of ferrite. Generally, this kind of morphology is present when the weld cooling rate is low and the Cr_eq_ to Ni_eq_ ratio decreases.

**Fig. 7:**
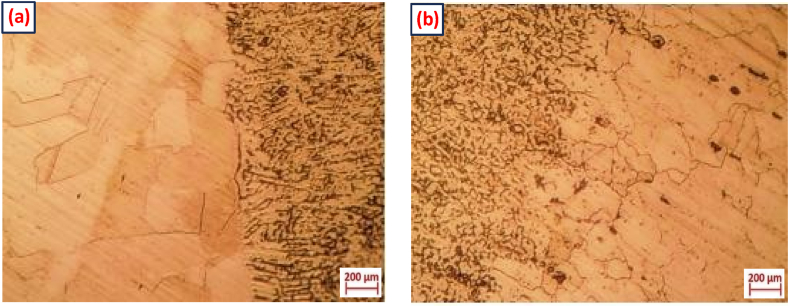
Optical micrograph of the HAZ **(a)** AISI 316L **(b)** AISI 321.

Fig. 7(a and b) shows the micrographs of HAZ on the AISI 316L and AISI 321 sides, respectively. It was noted that the HAZ did not exhibit any ditch structure, indicating no sensitization. The HAZ on the AISI 316L side of the weld joint was not sensitized, as it is a step structure (Fig. 7(a)). However, on the AISI 321 side of the weld joint (Fig. 7(b)), the step structure is shown. Since there was no ditch structure, it was inferred that the AISI 316L and AISI 321 weld joints' HAZ was not sensitized.

**Fig. 8:**
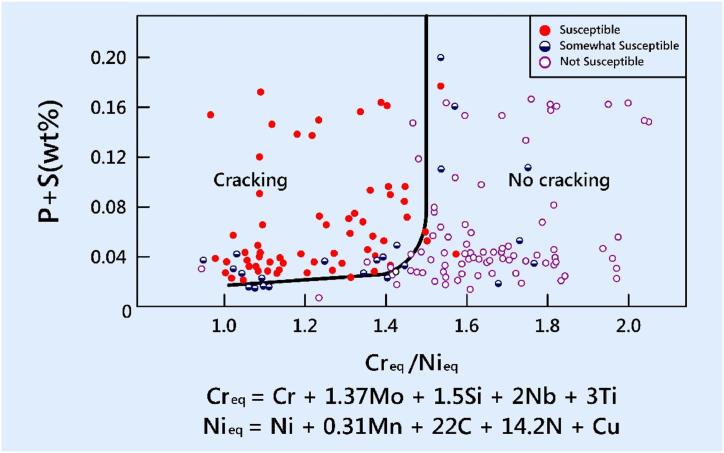
Suutala diagram.

The analysis of solidification cracking in the fusion zone was conducted using the Suutala Diagram. This specific diagram, depicted in Fig. 8, was developed through a thorough examination of various published articles on weld metal cracking in austenitic stainless steel (ASS).

The diagram effectively illustrates the importance of cracking susceptibility in ASS weld metal. Notably, as the levels of critical elements Cr_eq_ and Ni_eq_ surpass a certain threshold, the resistance to cracking displays a rapid increase [28]. For the specific dissimilar combination being studied, the susceptibility to cracking during solidification was determined by calculating the Cr_eq_ and Ni_eq_ values for the solidified weld. Furthermore, the Cr_eq_ and Ni_eq_ ratios were plotted on the Suutala diagram, and cracking behavior was investigated. Fig. 9 (a), Fig. 9 (b), Fig. 9 (c)(a–c) shows EDAX analysis along the center line of the weld. Table 3 shows Cr_eq_ to Ni_eq_ levels in the different zones of the fusion zone. From EDAX analysis for the present dissimilar combination, it is observed that the ratio of Cr_eq_ to Ni_eq_ is higher than the critical value in all possible locations along the center line of the weld. From Table 3, the higher ratio was obtained at the root of the weld.

**Fig. 9 (a):**
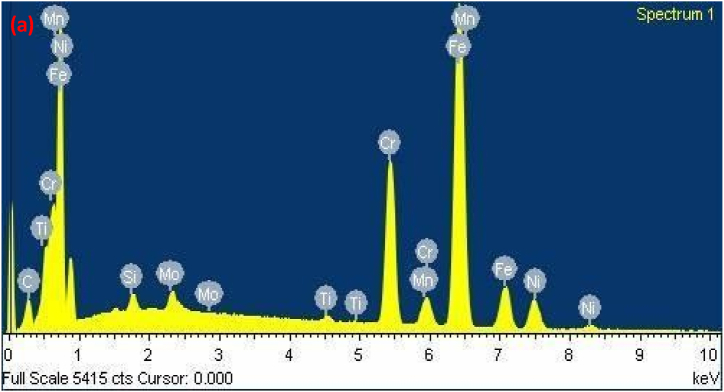
EDAX analysis of Root region of fusion zone.

**Fig. 9 (b):**
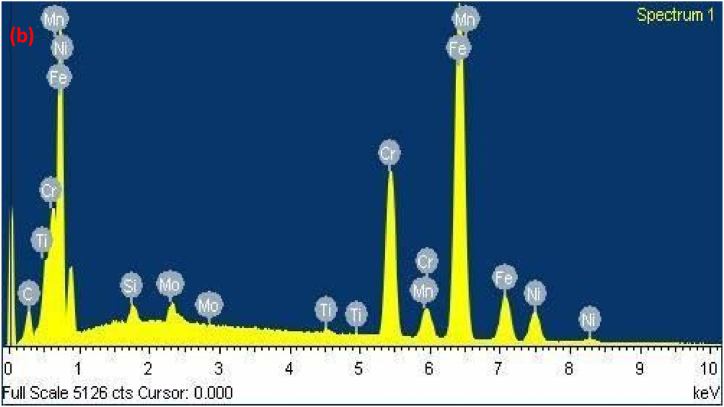
EDAX analysis of Middle region of fusion zone.

**Fig. 9 (c):**
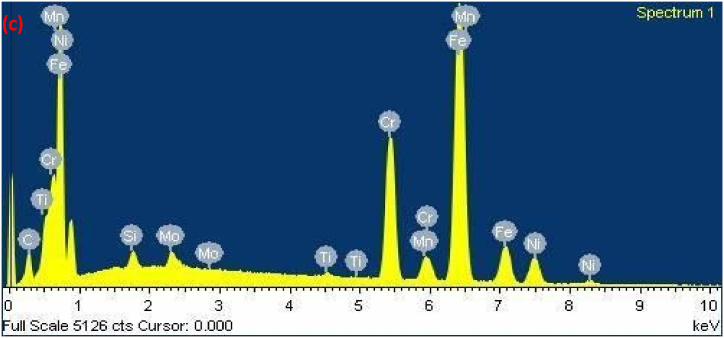
EDAX analysis of Crown region of fusion zone.

### 3.2 Fractography analysis

**Fig. 10:**
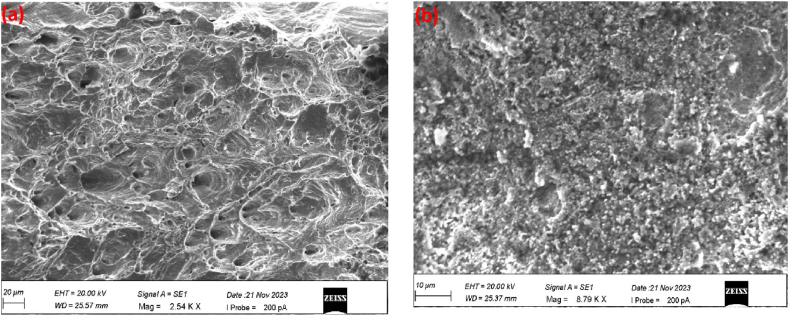
SEM of fracture sample (a) room temperature (b) cryogenic temperature.

SEM fractography of tensile specimens is shown in Fig. 10(a and b). These SEM images provide valuable insights into the failure mechanisms and differences in fracture surfaces between room temperature and cryogenic conditions. At room temperature, it was found that the joint typically exhibits ductile fracture characteristics with visible dimples or micro-voids on the fracture surfaces, as shown in Fig. 10 (a). At cryogenic temperatures, it was observed that the materials tend to become more brittle, and brittle fracture mechanisms were found, which is shown in Fig. 10 (b). However, at cryogenic temperatures, the increased brittleness may also be attributed to the propensity for intergranular fracture, where the fracture propagates along the grain boundaries rather than through the grains themselves. This phenomenon can be observed in Fig. 10(b) as distinct, straight fracture lines along the boundaries between grains (see Fig. 10(b)).

### 3.3 Mechanical testing

#### 3.3.1 Tensile testing

The tensile strength of the weldments was analyzed at ambient as well as cryogenic conditions. To achieve a proper evaluation of the results, three samples were tested in each condition. The testing in the ambient condition (303K) was performed at a constant strain rate of 2 × 10^−3^ s-1, whereas in the cryogenic condition (77K), the strain rate was set at 3 × 10^−4^ s^−1^. Table 4 illustrates the results of tensile tests conducted at ambient and cryogenic temperatures, respectively. The average tensile properties at ambient temperature were found to be UTS of 621 MPa, yield strength of 337 MPa, and percentage elongation of 47. On the other hand, average tensile properties at cryogenic conditions were found to be 1319 MPa, yield strength of 436 MPa, and percentage elongation of 39. The results showed that the test temperatures had a significant impact on the values of ultimate tensile strength. Similar observations were drawn by Hussain et al. [21] in plasma-arc-welded SS 316 in cryogenic conditions. While greater percentage elongation was seen at a test temperature of 303 K and good strength was revealed at a test temperature of 77 K.

#### 3.3.2 Microhardness testing

**Fig. 11:**
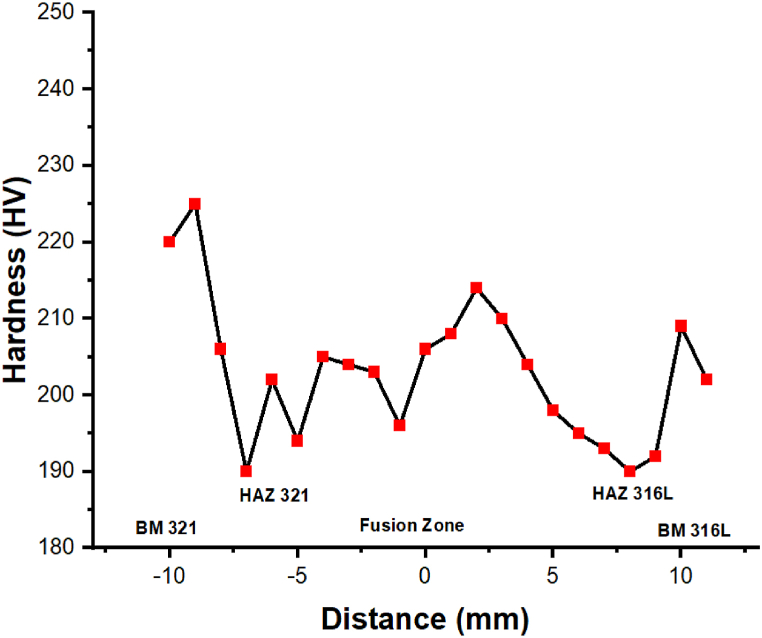
Graphical representation of Microhardness across the weldments.

The hardness variations across the weldments were analyzed using Vickers microhardness analysis. The microhardness assessment was carried out over 22 locations with a 1 mm interval between each point. Fig. 11 shows the graphical representation of microhardness across the weldments. From the figure, it is clear that the highest hardness was associated with the AISI 321 side. The fusion zone showed a large deviation in the profile (205 ± 10 HV). However, the lowest hardness was associated with the HAZ zones. Furthermore, from microstructural evidence, it is also observed that the lacy ferrite morphology has shown significant improvement in the hardness in the middle part of the weld joint.

## 4 Conclusions

1. The study successfully employed the PCGTAW technique to join AISI 316L and AISI 321 plates, achieving optimized process parameters. Microstructural analysis of the fusion zone revealed a complex morphology without any indication of solidification cracking, indicating the effectiveness of the welding process. Additionally, IGC testing demonstrated no sensitization in the Heat Affected Zone (HAZ), although variations in grain size were noted between AISI 316L and AISI 321.
2. On analyzing the tensile properties, it was observed that the testing environment significantly influences the tensile strength of the dissimilar welded joints. The average tensile properties at room temperature were found to be UTS of 621 MPa, highlighting the material's performance under ambient conditions. Conversely, average tensile properties at cryogenic conditions surged to as high as 1319 MPa, indicating a substantial enhancement in mechanical strength under low-temperature environments.
3. Microhardness analysis of the weldments revealed that the highest hardness was associated with the AISI 321 side. Notably, the fusion zone exhibited a considerable deviation in the hardness profile, with values averaging 205 ± 10 HV. Interestingly, the highest average hardness was observed in the middle part of the weld, suggesting localized variations in microstructure and mechanical properties.

Our future research focuses on investigating the effects of additional factors, such as welding speed and heat input, on weld quality and performance, which would provide deeper insights. Moreover, exploring advanced characterization techniques & impact test to understand the underlying mechanisms affecting tensile properties and hardness distribution would be beneficial.

## Funding

The authors thank 10.13039/501100002383King Saud University for funding this work through Re-searchers Supporting Project number (RSP2024R164), 10.13039/501100002383King Saud University, Riyadh, Saudi Arabia. This article was co-funded by the European Union under the REFRESH – Research Excellence For REgion Sustainability and High-tech Industries project number CZ.10.03.01/00/22_003/0000048 via the Operational Programme Just Transition and has been done in connection with project Students Grant Competition SP2024/087, “Specific Research of Sustainable Manufacturing Technologies” financed by the Ministry of Education, Youth and Sports and Faculty of Mechanical Engineering VŠB-TUO.

## CRediT authorship contribution statement

**Tejas Patil:** Writing – review & editing, Writing – original draft, Software, Formal analysis, Data curation. **Ajit Bhosale:** Supervision, Resources, Methodology, Investigation, Formal analysis, Conceptualization. **S.G.K. Manikandan:** Visualization, Software, Resources, Project administration, Investigation. **Bibin Jose:** Validation, Software, Methodology, Investigation, Funding acquisition. **Mithul Naidu:** Writing – review & editing, Writing – original draft, Software, Project administration, Methodology, Investigation, Formal analysis, Data curation. **Sachin Salunkhe:** Writing – review & editing, Validation, Supervision, Conceptualization. **Robert Cep:** Writing – review & editing, Visualization, Resources, Funding acquisition, Formal analysis. **Emad Abouel Nasr:** Writing – review & editing, Writing – original draft, Validation, Supervision, Methodology, Funding acquisition.

## Declaration of competing interest

The authors declare the following financial interests/personal relationships which may be considered as potential competing interests:Dr. Sachin Salunkhe reports financial support was provided by 10.13039/501100002383King Saud University. If there are other authors, they declare that they have no known competing financial interests or personal relationships that could have appeared to influence the work reported in this paper.
